# Interface agent modified by ternary polymers for ceramic tile of old walls based on grey relational analysis

**DOI:** 10.1371/journal.pone.0320517

**Published:** 2025-04-03

**Authors:** Guanji Lyu, Mingxin Zheng, Tao Ji, Yalin Ma

**Affiliations:** 1 College of Transportation Engineering, East China Jiaotong University, Nanchang, Jiangxi, China; 2 Department of Management Engineering, Fujian Business University, Fuzhou, Fujian, China; 3 College of Civil Engineering, Fuzhou University, Fuzhou, Fujian, China; Instituto Federal do Espírito Santo: Instituto Federal de Educacao Ciencia e Tecnologia do Espirito Santo, BRAZIL

## Abstract

The Interface Agent for Old Wall Tiles (IAWT) is an environmentally friendly material. However, its application in engineering is limited by its tendency to lose adhesion under harsh conditions. Factors such as freeze-thaw cycles, wind vibrations, rainfall, and thermal stress negatively affect the bond strength of IAWT. This study investigated the effects of PTB emulsion on various properties of IAWT, including bond strength, freeze-thaw resistance, wind vibration resistance, compressive-flexural ratio, water absorption, and microstructure. Orthogonal experiments were conducted to examine the impact of three key factors on the macroscopic properties of IAWT. The results indicated that increasing the ratio of PTB emulsion to water (P/W) improved bond strength under normal environmental conditions and enhanced freeze-thaw and wind vibration resistance. Furthermore, a higher P/W ratio reduced both the compressive-flexural ratio and water absorption. The addition of PTB emulsion slowed the hydration reaction, increased the number of micropores, and reduced the cumulative pore volume. Furthermore, it enhances the bond strength between the coating and the base layer, thereby increasing the durability of the renovation project for old walls. These enhancements yield positive economic and environmental benefits.

## Introduction

**Fig 1:**
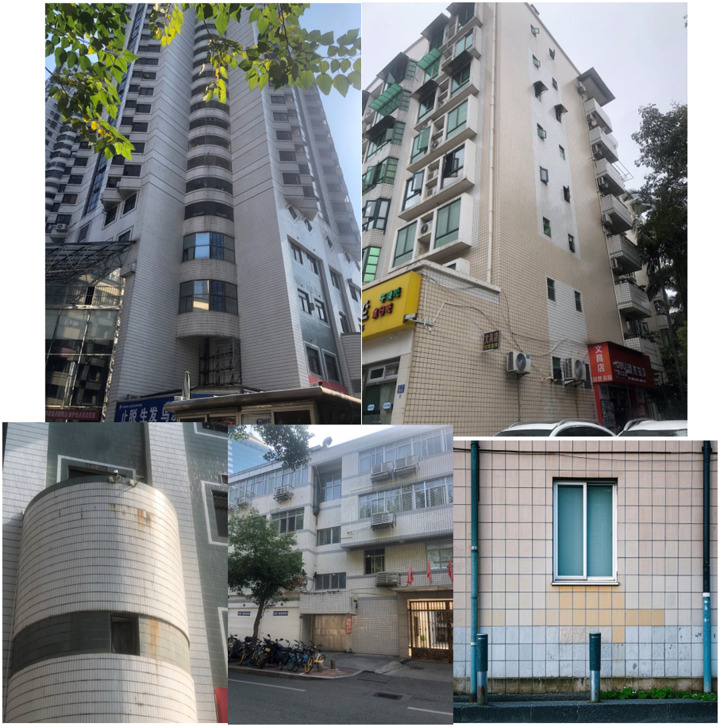
Aged buildings with tiled exteriors.

With rapid urbanization in China, a large number of buildings have been constructed in recent years to meet the demands of social and economic development. However, as these buildings age and aesthetic preferences evolve, attention is increasingly turning towards strengthening, maintenance, and renovation [1]. Historically, as illustrated in Fig 1, tiles and mosaics were widely employed for the decoration of exterior walls [2]. The buildings depicted in Fig 1 are generally between 15 and 35 years old. As these buildings age, not only will their overall appearance decline, but safety risks will also emerge [3–5]. Consequently, the development of a safe, efficient, and environmentally friendly method for renovating old wall tiles has become an urgent challenge for researchers.

More than 90% of developed countries utilize modern coatings for exterior wall decoration [6–7]. Similarly, many homeowners in China seek to replace old wall tiles with paint. However, the smooth surfaces of ceramic tiles on the exterior walls of older buildings, combined with exposure to wind, rain, and solar radiation, can result in poor adhesion, pitting, and even paint peeling. For instance, in Beijing, the lowest temperature can drop to -16°C, while the highest can reach 41°C, resulting in a temperature variation of 57°C. The average wind speed in winter is approximately 4.7 m/s, with windy conditions persisting for five months. Many regions in China have experienced significant cycles of freezing and thawing, particularly in high-altitude areas where wind speeds may be elevated. To address this issue, some researchers advocate for the chemical functionalization of ceramic tile surfaces using coupling agents to enhance adhesion with polymer-modified mortar [8]. Additionally, several studies have evaluated the effects of polymer-modified interfacial agents on the complex interactions at the mortar-tile interface [9–11]. However, they did not consider the effects of freeze-thaw and wind vibration.

Polymers play a significant role in contemporary material applications [12–15], particularly within the construction industry [16,17]. Researchers have utilized styrene-butadiene rubber (SBR) latex to enhance the water and corrosion resistance of concrete [18–21]. Epoxy resin latex improves the adhesion and impermeability of mortar [22–24]. Ethylene vinyl acetate (EVA) latex enhances the mechanical and hydration properties of mortar [10,25–28]. Acrylic latex-modified mortar exhibits excellent mechanical properties and corrosion resistance [29–30]. Organic silicon-modified mortar shows improved workability, mechanical properties, physical characteristics, and internal pore structure [31–34]. Additionally, various polymer composite materials exert differing degrees of influence on concrete and mortar [35–38]. However, current research on polymer-modified cement-based materials has yet to address the renovation of old wall tiles. Furthermore, no reports have been found on the bond strength of IAWT under freeze-thaw and wind vibration.

In this paper, we introduce the terpolymer (PTB) to investigate the bond strength, compressive-flexural ratio, water absorption, freeze-thaw resistance, and wind vibration resistance of IAWT. The macroscopic performance is complemented by microscopic analyses, including X-ray Diffraction (XRD), Mercury Intrusion Porosimetry (MIP), and Environmental Scanning Electron Microscopy (ESEM). The research employs preliminary mix proportions and orthogonal experiments, followed by grey relational analysis of the results. Furthermore, this study serves as a practical implementation of the energy-saving and environmental protection initiatives advocated by the government.

## Materials and methods

### Raw materials

**Fig 2:**
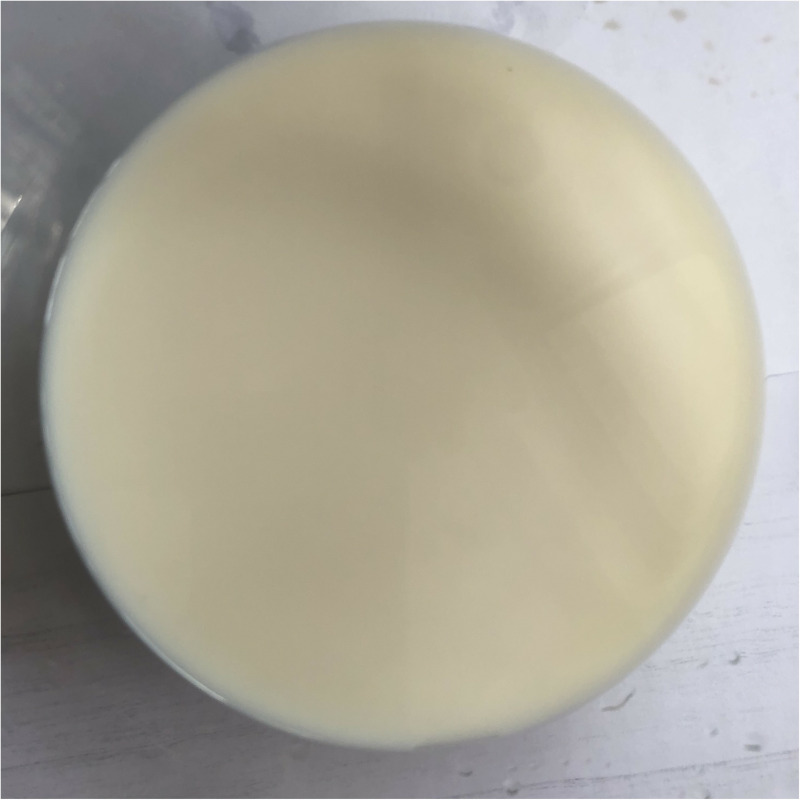
The phase state of the PTB emulsion.

The cement employed in this study is 42.5R ordinary Portland cement, produced by Zhejiang Hongshi Cement Co., Ltd. The primary chemical constituents of the cement are detailed in Table 1. The sand utilized in this project complies with the specifications of GB/T 17671–1999 and is sourced from Xiamen Aisiou Standard Sand Co., Ltd. The relevant indicators of standard sand are shown in Table 2. The PTB emulsion used in this application is a ternary polymer composed of vinyl chloride, ethylene, and vinyl ester, manufactured by Kingdom of Belgium Fine Chemical Industry Co., Ltd. The technical parameters of the PTB emulsion are presented in Table 3, and its phase diagram is illustrated in Fig 2.

**Table 1:** Main chemical components of cement.

Chemical component	CaO	SiO_2_	Al_2_O_3_	Fe_2_O_3_	MgO	SO_3_	Na_2_O	TiO_2_	MnO
Content (%)	59.65	20.41	8.59	3.36	1.60	3.45	0.73	0.37	0.24

**Table 2:** Relevant indicators of standard sand.

SiO_2_	Loss on ignition	Moisture content	Clay content	Cl content	Floating objects	Sieve diameter and cumulative sieve residue					
						2mm	1.6mm	1.0mm	0.5mm	0.16mm	0.08mm
98%	<0.4%	<0.18%	<0.18%	<0.007%	<0.001%	0	7%	33%	67%	87%	100%

**Table 3:** Technical parameters of PTB emulsion.

Solid content (%)	Viscosity (MPa·s)	TG^a^ (°C)	pH	MFT^b^ (°C)	Density (g/cm³)
52 ± 1	80 ± 20	7	7 ～ 9	7	1.1

^a^TG signifies the glass transition temperature.

^b^MFT denotes the lowest film-forming temperature.

### Mix proportion and sample preparation

The preliminary mix proportions, as illustrated in Table 4, have been formulated to elucidate the impact of PTB emulsion on the interfacial agent. Notably, the ratio of PTB emulsion to water (P/W) has been varied from 1:1–1:5 (by volume). It is important to emphasize that the masses of cement, sand, and solution remain constant throughout the experiment. When the P/W ratio is 0, it signifies the absence of PTB emulsion, resulting in the mixture transforming into conventional mortar. Furthermore, the term “solution” refers to the combination of PTB emulsion and water, where the total mass remains unchanged while the P/W ratio varies.

**Table 4:** Preliminary mix proportion.

Group	Cement (g)	Sand (g)	Solution(g)	PTB emulsion (g)	Water (g)	PTB emulsion: Water (P/W)
D0	520	1300	234	0	234	0
D1	520	1300	234	42.2	191.8	1:5
D2	520	1300	234	50.5	183.5	1:4
D3	520	1300	234	62.8	171.2	1:3
D4	520	1300	234	83.0	151.0	1:2
D5	520	1300	234	122.6	111.4	1:1

Table 5 presents the orthogonal experimental mix proportions, which are employed for the further optimization of the overall performance of IAWT. By conducting an orthogonal experiment, we can analyze the impact of various factor levels on the performance of IAWT.

**Table 5:** Orthogonal experimental mix proportion.

Level	A	B	C
	P/W	Solution to cement ratio (SO/C)	Cement to sand ratio (C/S)
1	1:5	0.40	1:1.0
2	1:3	0.45	1:1.5
3	1:1	0.50	1:2.0

**Fig 3:**
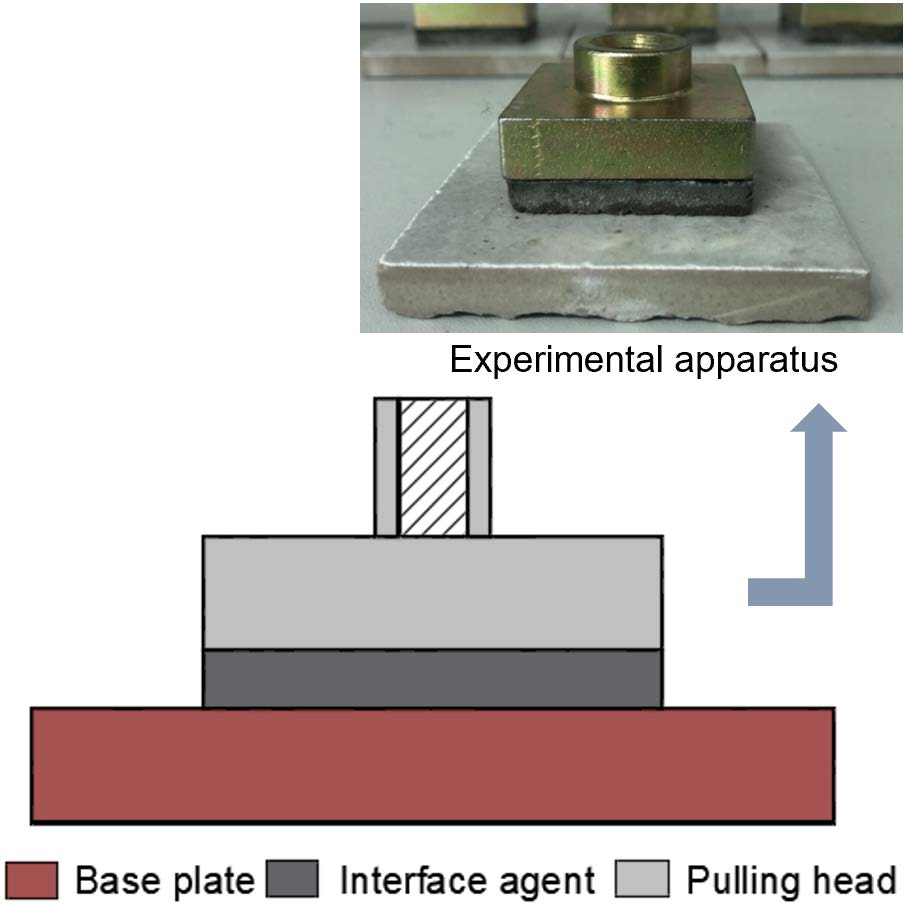
Schematic diagram of bond strength test specimens.

The bond strength test specimen is illustrated in Fig 3. The specimen is cured for 7 days and 14 days under standard conditions. The IAWT used for bond strength testing in normal environmental conditions has a thickness of (10 ± 1) mm. For bond strength specimens designated for freeze-thaw and wind vibration testing, the IAWT thickness is (10 ± 1) mm and (5 ± 1) mm, respectively.

The specimens utilized for the compressive-flexural ratio test have dimensions of 40 mm ×  40 mm ×  160 mm. These specimens were cured for 28 days under standard conditions. Similarly, the water absorption test specimens share the same dimensions and curing duration as the compressive-flexural ratio specimens.

In the MIP (Mercury Intrusion Porosimetry) test, specimens cured for 28 days are crushed, and bean-shaped samples with an approximate diameter of 3 mm are selected. To prepare the samples, any powdery impurities on the surface are first removed using a wash ball, followed by the termination of hydration treatment with an isopropyl alcohol solution. After the hydration treatment is terminated, these samples are dried in a vacuum oven at 45 °C.

For XRD (X-ray Diffraction) testing, the samples are ground into a powder using an agate mortar and then sieved through a 75 μm sieve. In the ESEM (Environmental Scanning Electron Microscopy) experiments, the interfacial regions that have reached a curing age of 28 days are crushed. The samples are subsequently immersed in anhydrous ethanol to halt the hydration process. Following this, the samples are dried in a vacuum oven at 45 °C. Prior to testing, the sections are gold-plated using a high-pressure ion sputtering apparatus.

### Test methods

The bond strength testing method follows DL/T 5126–2021. The HC-2000A bond strength tester is used as the testing apparatus. The freeze-thaw resistance test was conducted using the TR-CLD freeze-thaw cycle machine, manufactured by Shanghai Concrete Instrument Equipment Co., Ltd. The test method adhered to the specifications outlined in GB/T 50082–2009. Specimens that have been cured for 7 days are removed and soaked in water at (20 ±  2) °C for 18 hours, then placed in a refrigerator at (-20 ±  3) °C and maintained at a constant temperature for 3 hours. Afterwards, they are taken out and placed in a high-temperature chamber at (50 ±  3) °C, where they are kept at a constant temperature for 3 hours. Each cycle lasts for 24 hours, with a total of 10 cycles.

The test for wind vibration resistance was conducted in accordance with the testing method described in Appendix A of T/CECS901–2021. A suction-type electromagnetic vibration table was utilized, with a vertical vibration frequency of 50 Hz, a vertical vibration amplitude of 3 mm, and a vibration duration of 30 minutes.

The compressive-flexural ratio test was conducted using the YAW-300C anti-bending and compression testing machine. Water absorption was measured with the LQ-C100001 electronic scale. All testing methods adhered to the specifications outlined in GB/T 17671–1999.

The Mercury Intrusion Porosimetry (MIP) test is employed to characterize the pore structure of the samples. The samples are placed inside a glass tube and evacuated using a vacuum pump. Subsequently, mercury is injected into the glass tube. The test consists of a low-pressure assessment ranging from 0 psi to 25 psi, followed by a high-pressure assessment ranging from 25 psi to 60,000 psi.

X-ray diffraction (XRD) analysis of the samples was conducted using an X/Pert Pro MPD X-ray diffractometer (DY5261/Xpert3, CEM, USA). The scanning angles (2θ) ranged from 5° to 75° at a rate of 0.01313° per second and a step size of 0.02°. The analysis utilized Cu Kα radiation (λ =  1.54 Å) at 40 kV and 40 mA to identify variations in the crystalline phase.

The Environmental Scanning Electron Microscopy (ESEM) experiments were conducted using the Quanta 250 scanning electron microscope manufactured by FEI (USA). This microscope utilizes a tungsten filament as its electron beam source.

## Results

**Fig 4:**
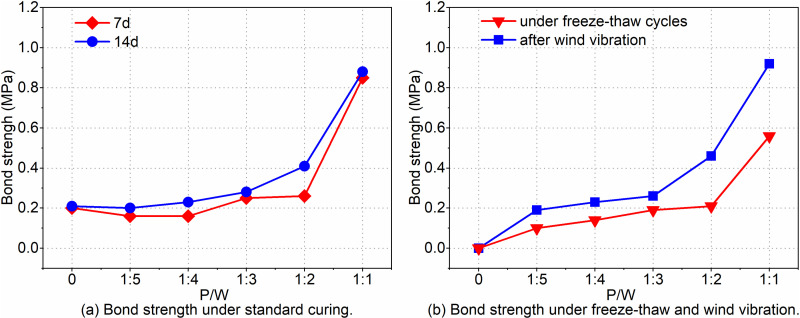
Bond strength. (a) Bond strength under standard curing. (b) Bond strength under freeze-thaw and wind vibration.

Fig 4(a) presents the bond strength results at 7 d and 14 d. According to the 7d bond strength curve, the bond strength generally increases with an increase in PTB emulsion content. Notably, when the P/W ratio exceeds 1:2, there is a sudden increase in bond strength. At a P/W ratio of 1:1, the bond strength measures 0.85 MPa. The 14d bond strength curve indicates that the bond strength of the Interface Agent for Old Wall Tiles (IAWT) with the PTB additive surpasses that of ordinary mortar. When the P/W ratio is less than 1:2, the bond strength of IAWT exhibits slower growth. However, when the P/W ratio exceeds 1:2, there is a significant improvement in the bond strength of IAWT, reaching 0.88 MPa at a P/W ratio of 1:1. At this ratio, the bond strength at 7 d and 14 d is 2.3 times and 1.8 times higher, respectively, compared to the strength at a ratio of 1:2. While the bond strength at 14 d is consistently higher than at 7 d, the rate of increase is relatively small. At P/W ratios of 0, 1:5, and 1:4, bond failure primarily occurs at the interface between the interface agent and the substrate, with only a small amount of the interface agent adhering to the substrate surface. At a P/W ratio of 1:3, some damage is observed within the interfacial agent layer, while additional damage occurs at the substrate interface. In contrast, at P/W ratios of 1:2 and 1:1, the damage predominantly takes place within the interfacial agent, resulting in a rough damage surface.

Fig 4(b) illustrates the bond strength after wind vibration and freeze-thaw cycles under different P/W conditions. Following freeze-thaw cycles, the bond strength of ordinary mortar approaches nearly zero. In contrast, as the P/W ratio increases, the bond strength of IAWT with the PTB emulsion additive demonstrates improvement after freeze-thaw cycles. At a P/W ratio of 1:1, the maximum bond strength after freeze-thaw cycles reaches 0.56 MPa, which is approximately 1.7 times higher than that at a P/W ratio of 1:2. After conducting the wind vibration test, it was observed that the bond strength of ordinary mortar was nearly negligible. In contrast, the bond strength of IAWT, when combined with the PTB emulsion additive, increased with higher P/W ratios. Specifically, at a P/W ratio of 1:1, the maximum bond strength recorded was 0.92 MPa, which represents a significant increase of 0.73 MPa compared to the bond strength observed at a P/W ratio of 1:5. Conversely, at the P/W ratio of 1:3, the bond strength experienced only a slight increase of 0.07 MPa compared to the ratio of 1:5.

**Fig 5:**
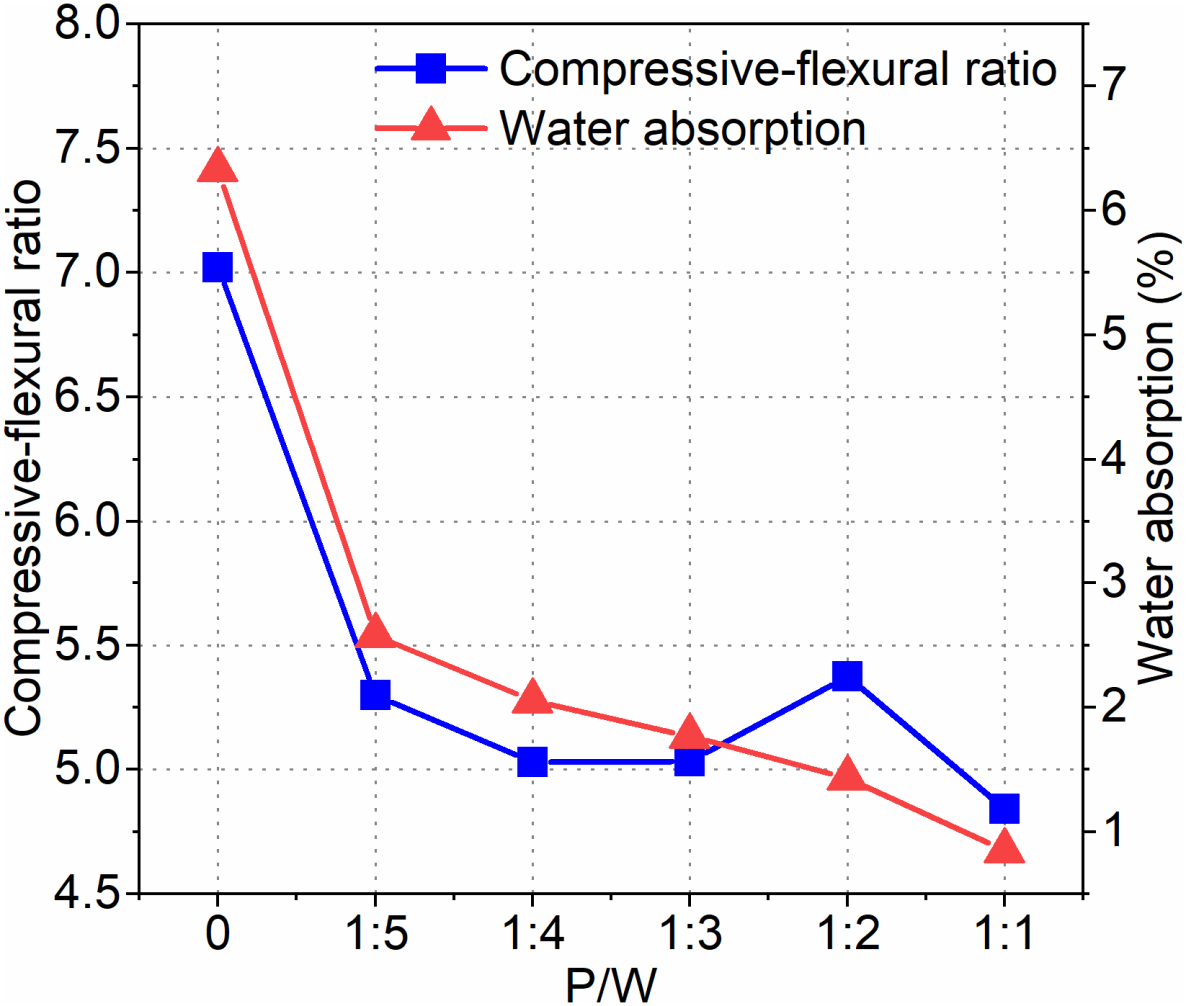
Compressive-flexural ratio and water absorption.

The compressive-flexural ratio and water absorption rate under different P/W ratios are depicted in Fig 5. When the PTB emulsion content is zero, the compressive-flexural ratio reaches its maximum value of 7.1. However, the addition of PTB emulsion significantly reduces the compressive-flexural ratio. Notably, a significant change occurs when the P/W ratio reaches 1:2. At a P/W ratio of 1:1, the minimum compressive-flexural ratio recorded is 4.84. When comparing the P/W ratios of 1:5 and 1:1, the compressive-flexural ratio decreases by approximately 25.4% and 31.8%, respectively, compared to the blank group. Additionally, the water absorption rate of IAWT decreases as the P/W ratio increases. At a P/W ratio of 1:1, the water absorption rate of IAWT is only 0.85%. Compared to the P/W ratios of 0 and 1:5, the water absorption rate at the P/W ratio of 1:1 decreases by 5.48% and 1.73%, respectively.

**Fig 6:**
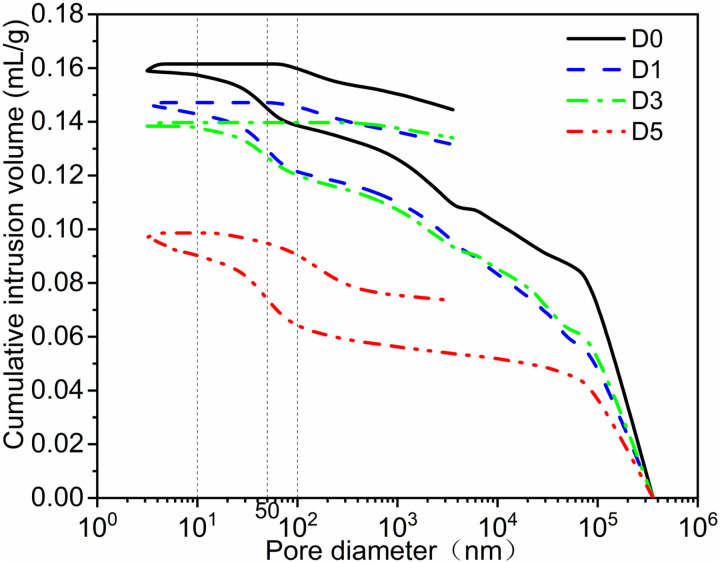
Cumulative pore volume curve.

**Fig 7:**
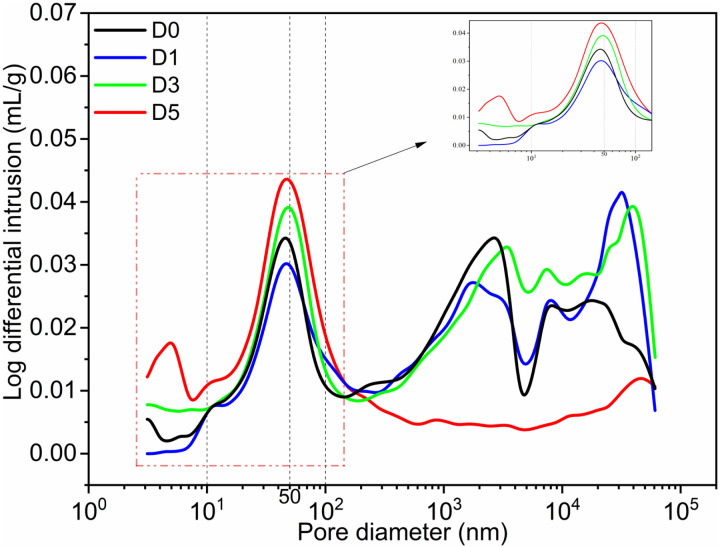
Differential curve of pore distribution.

**Fig 8:**
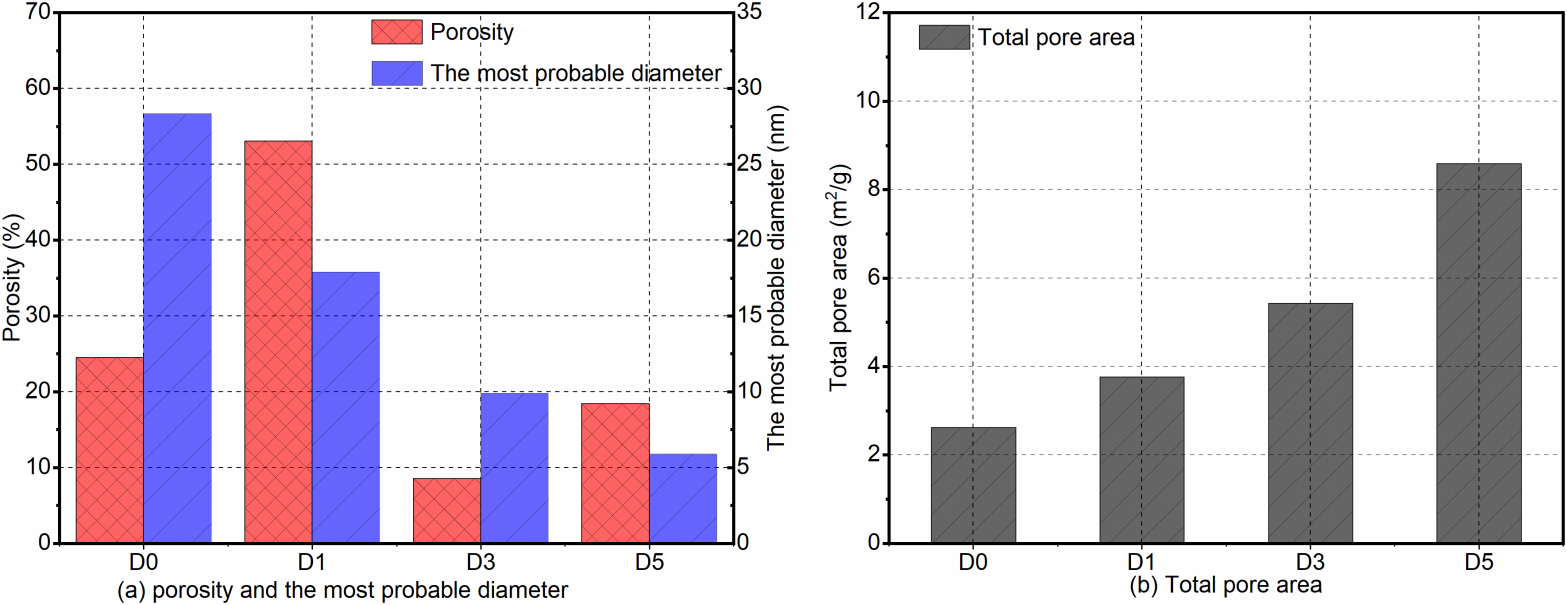
Porosity, most probable pore size, and total pore area bar chart. (a) The porosity and the most possible diameter of the D0, D1, D3, and D5 groups are presented. (b) Total pore area of the D0, D1, D3, and D5 groups are presented.

Figs 6-7 illustrate the cumulative pore volume curve and the differential pore distribution curve, respectively. Fig 8 presents a bar chart depicting porosity, the most probable pore size, and the total pore area. Among the groups, the D3 group exhibits the lowest porosity at 8.61%, while the D5 group demonstrates higher porosity, reaching 18.44%. The most probable pore size for the D5 group is 5.91 nm, compared to 9.89 nm for the D3 group, indicating a difference of 3.98 nm, with the D3 group having the larger pore size. The total pore area for the D5 group is 8.565 m²/g, whereas the D0 group measures 2.624 m²/g for total pore area. From Fig 6, it is evident that the D5 group has the smallest cumulative pore volume for apertures below 100 nm and the least cumulative pore volume for apertures above 100 nm, in contrast to the D0 group. Fig 7 further supports this observation with the differential pore distribution curve.

**Fig 9:**
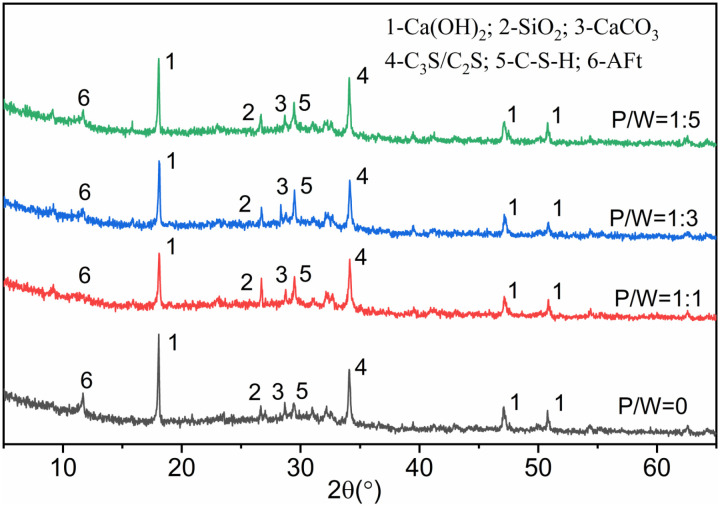
XRD analysis diagram of cement paste sample.

X-ray diffraction (XRD) analysis was performed on the cement slurry samples, with results presented in Fig 9. In the groups containing PTB emulsion, the peak intensities of Ca(OH)₂ and AFt diffraction were significantly lower compared to the control group. Additionally, the peak intensity of C₂S/C₃S was also slightly reduced. These findings indicate that the addition of PTB emulsion results in a decrease in Ca(OH)₂ levels and a delay in the hydration of C₂S/C₃S. It was observed that the intensities of the diffraction peaks for Ca(OH)₂, AFt, and C₂S/C₃S increased as the content of PTB emulsion decreased. This phenomenon can be attributed to the bonding forces generated by the carboxyl groups in the polymer, which complex with Ca² ⁺ ions on the surface of cement particles. Consequently, incomplete contact between the cement particles and water occurs, hindering the migration of ions in the cementitious liquid phase and slowing the reaction rate of cement hydration. The results demonstrate that the bond strength of Interface Agent for Old Wall Tiles (IAWT) mixed with PTB is significantly greater than that of conventional mortar. Furthermore, bond strength increases proportionally with the amount of polymer used. This finding suggests that in polymer mortar, the quantity of hydration products cannot be solely relied upon as a definitive indicator of strength levels. Instead, bond strength is influenced by a combination of the inherent properties of the organic material, hydration products, and microstructure.

**Fig 10:**
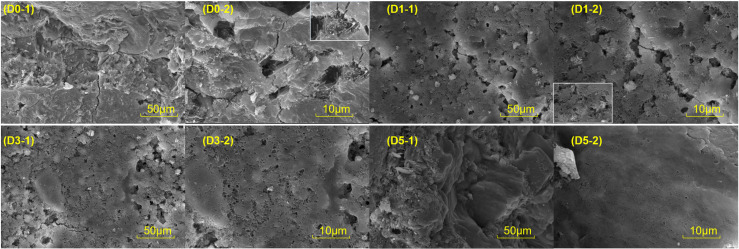
ESEM Micromorphology.

The microstructure observed after conducting ESEM testing is presented in Fig 10. It is important to note that the microstructure plays a crucial role in determining the macroscopic mechanical properties. Upon analyzing the image, it becomes evident that the D0 group contains a substantial number of interconnected pores resulting from crack formation. Furthermore, the texture appears porous due to inadequate hydration of numerous cement particles, which leads to low bond strength, a high compressive-flexural ratio, and a high water absorption rate. In contrast, the D1 group exhibits significant improvements in crack formation and a reduction in the size of large pores. The D3 group shows a notable decrease in both pore size and crack formation. Finally, the cementitious matrix in the D5 group retains its integrity, indicating a compact and dense structure.

**Fig 11:**
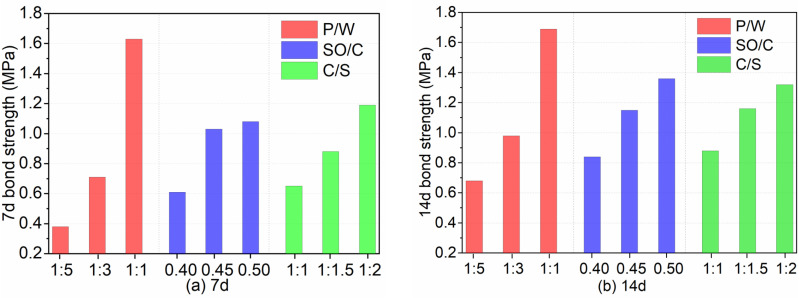
Bond strength. (a) The 7 d bond strength is evaluated under various influencing factors. (b) The 14 d bond strength is evaluated under various influencing factors.

**Fig 12:**
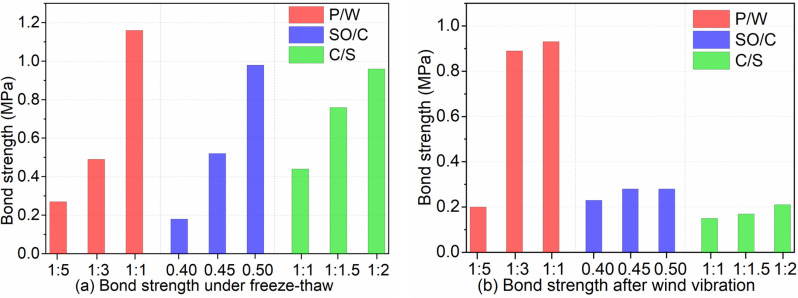
Bond strength after freeze-thaw and wind vibration. (a) Bond strength under freeze-thaw cycles. (b) Bond strength after wind vibration.

**Fig 13:**
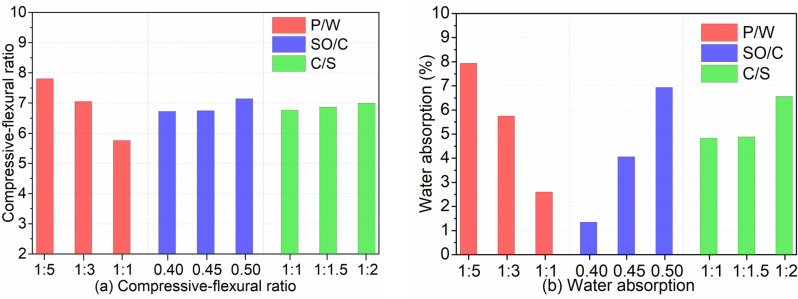
Compressive-flexural ratio and water absorption. (a) The compressive-flexural ratio under different influencing factors. (b) The water absorption under different influencing factors.

Fig 11 illustrates the influence of three factors on bond strength at 7 d and 14 d. Fig 12 depicts the effects of these factors on bond strength after freeze-thaw cycles and wind vibration. Fig 13 presents the impact of the three factors on the compressive-flexural ratio and water absorption. As shown in Fig 11, the bond strength at 7 d and 14 d decreases with increasing P/W and SO/C ratios, but increases with decreasing C/S ratios. Notably, the bond strength at 14 d has improved to varying degrees compared to that at 7 d, with the highest growth rate being 78.9% and the lowest at 3.7%. Additionally, Fig 12 indicates that bond strength after freeze-thaw cycles and wind vibration decreases with increasing P/W and SO/C ratios, while it increases with decreasing C/S ratios. Furthermore, the bond strength after freeze-thaw and wind vibration is reduced to varying degrees compared to the strength under standard curing conditions. Moreover, Fig 13 shows that the compressive-flexural ratio decreases with increasing P/W and slightly increases with increasing SO/C. Conversely, the water absorption rate significantly decreases with increasing P/W and decreases with increasing SO/C. The compressive-flexural ratio and water absorption slightly increase with decreasing C/S. The following bar chart presents the average experimental results for each factor at different levels. For instance, the variation in bond strength at 7 d with respect to P/W is calculated by averaging the test results at levels of 1:1, 1:3, and 1:5, respectively.

## Discussion

### Macro performance and micro mechanism

Bond strength is a critical aspect of the interfacial agent’s performance, as it plays a vital role in preventing the decorative layer from detaching and posing a threat to community safety. This study aims to examine three types of bond strength: normal environment, freeze-thaw cycle, and wind vibration (Fig 4). To analyze bond strength, we introduce the concept of the residual strength ratio, represented by Equation (1).


$$R=\frac{f_{1}}{f_{0}}\times 100\%$$ (1)

$f_{1}$ is the bond strength after freeze-thaw cycles or wind vibration. $f_{0}$ is the corresponding bond strength before freeze-thaw cycles or wind vibration. R is the residual strength ratio.

**Fig 14:**
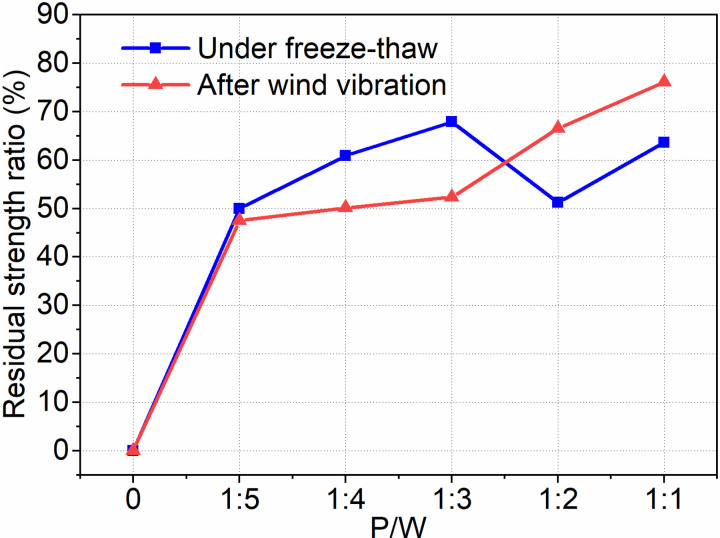
Residual strength ratio.

Fig 14 demonstrates that the residual strength of Interface Agent for Old Wall Tiles (IAWT) with PTB emulsion is higher than that of the blank group after undergoing freeze-thaw cycling and wind vibration. This indicates that the PTB emulsion-modified IAWT has the capability to withstand challenging environmental conditions. The maximum residual strength under freeze-thaw conditions is achieved at a P/W ratio of 1:3, reaching 67.9%. In comparison, the P/W ratio of 1:1 only experiences a slight decrease of 4.3%. Furthermore, as the dosage of PTB emulsion increases, the residual strength after wind vibration also rises. Notably, when the P/W ratio is 1:1, the residual strength after wind vibration reaches its peak. When the PTB emulsion dosage increases to 1:1, the polymer particles in the emulsion gradually aggregate and fuse with the evaporation of water, forming a continuous polymer film in the mortar. This polymer film exhibits good bonding properties, filling the pores within the mortar, enhancing cohesion, and creating a strong bonding interface between the mortar and the bonded material.

The smaller the compressive-flexural ratio, the better the toughness. The external wall is subjected to alternating dry and wet conditions as well as temperature fluctuations. Improved toughness can help prevent cracking under such circumstances. According to Fig 5, the compressive-flexural ratio is lowest when the P/W ratio is 1:1. Furthermore, with a P/W ratio of 1:1, the water absorption rate is only 0.85%, effectively preventing rainwater infiltration. Fig 10 also indicates that when the P/W ratio is 1:1, the microstructure is the most compact, and hydration is the most complete. In contrast, the blank group exhibits more cracks and larger pore sizes.

**Fig 15:**
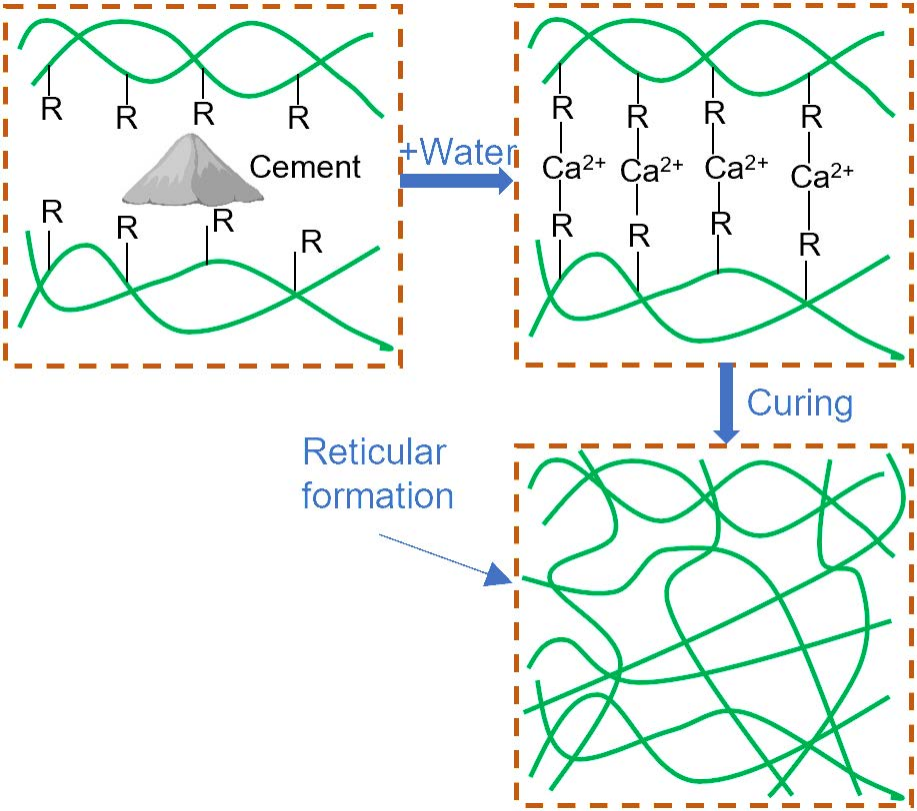
PTB reinforced cement hydration process diagram. (a) Combine the PTB emulsion with cement. (b) Calcium ions in the cement react with the PTB emulsion. (c) The PTB emulsion gradually solidified and intermingled with hydration products to form a network structure.

The process of enhancing Interface Agent for Old Wall Tiles (IAWT) with PTB emulsion is illustrated in Fig 15. From Figs 15(a) and 15(b), it is evident that the incorporation of PTB emulsion into the cement mortar produces a chemical reaction that improves the performance of IAWT. As shown in Fig 15(c), over time, the PTB emulsion gradually solidifies and intertwines with the hydration products, ultimately forming a complex and robust spatial framework-matrix network structure within the polymer mortar. The elastic modulus of this specific structural system is significantly lower than that of cement stone, yet it exhibits good toughness and superior bond performance [39–43]. The structure becomes denser, and the bond area increases, which contributes to its strong performance after wind vibration and freeze-thaw cycles. Additionally, PTB emulsion promotes the formation of micropores in the cement matrix, thereby enhancing the frost resistance of the mortar. These microporous structures also hinder the infiltration of water molecules, further improving the waterproofing performance.

### Grey relational analysis

Grey relational analysis is a fundamental principle that involves comparing the geometric relationships of data sequences within a system [44–46]. Its purpose is to determine the level of correlation among various technical indicators. The degree of correlation is assessed by examining how closely the geometric shapes of the sequence curves align. When the curves are closely aligned, the correlation between them is higher, and vice versa. To calculate the correlation coefficient, all data must have a unified dimension. Therefore, it is necessary to perform dimensionless processing on all sequences prior to the calculation.


$${\chi^{\prime }}_{\text{i}}\left(k\right)=\left(\chi_{i}\left(1\right),\chi_{i}\left(2\right),\cdot \cdot \cdot ,\chi_{i}\left(m\right)\right)$$ (2)

$$\chi_{\text{i}}\left(k\right)=\frac{{\chi^{\prime }}_{i}\left(k\right)}{\frac{1}{m}\sum_{k=1}^{m}{\chi^{\prime }}_{i}\left(k\right)}$$ (3)

The original data in column ${\chi^{\prime }}_{\text{i}}\left(k\right)$ is used as the input. By applying formula (3) to the original data in column ${\chi^{\prime }}_{\text{i}}\left(k\right)$, we obtain a new sequence, $\chi_{i}\left(k\right)$. This new sequence, $\chi_{i}\left(k\right)$, is then utilized in formula (4 ~ 5) to calculate the desired result.


$$\zeta_{i}(k)=\frac{\underset{i}{\min}\underset{k}{\min}\left|\chi_{0}\left(k\right)-\chi_{i}\left(k\right)\right|+\rho \cdot \underset{i}{\max}\underset{k}{\max}\left|\chi_{0}\left(k\right)-\chi_{i}\left(k\right)\right|}{\left|\chi_{0}\left(k\right)-\chi_{i}\left(k\right)\right|+\rho \cdot \underset{i}{\max}\underset{k}{\max}\left|\chi_{0}\left(k\right)-\chi_{i}\left(k\right)\right|}$$ (4)

$$\gamma_{0\text{i}}=\frac{1}{m}\sum_{k=1}^{m}\zeta_{i}\left(k\right)$$ (5)

In the provided formulas, $\chi_{0}\left(k\right)$ represents the processed reference sequence. $\chi_{i}\left(k\right)$ represents the processed factor sequences. $\underset{i}{\min}\underset{k}{\min}\left|\chi_{0}\left(k\right)-\chi_{i}\left(k\right)\right|$ represents the two-level minimum range. $\underset{i}{\max}\underset{k}{\max}\left|\chi_{0}\left(k\right)-\chi_{i}\left(k\right)\right|$ represents the two-level maximum range. $\rho \in \left[0,1\right]$ represents the resolution coefficient. $\zeta_{i}\left(k\right)$ represents the correlation coefficient. And $\gamma_{0i}$ represents the degree of correlation.

According to the grey relational analysis method, the 14d bond strength of the nine groups is used as the reference sequence, while the corresponding orthogonal experimental factors are considered as the comparative sequences. By applying the grey relational formula, the correlation coefficients can be calculated. It is evident that the correlation coefficients for the three factors affecting the 14d bond strength are 0.7197, 0.7049, and 0.6476, respectively. The P/W factor exhibits the highest degree of correlation with the 14d bond strength. This same method can be employed to determine the correlation coefficients of the three factors with other performance indicators.


$$\zeta_{\text{i}}=\left[\begin{array}{ccccccccc} 0.9106 & 0.7354 & 0.8694 & 0.8054 & 0.7092 & 0.6800 & 0.5795 & 0.7264 & 0.4610\\ 0.9106 & 0.9002 & 0.4202 & 0.8137 & 0.7092 & 0.4795 & 0.7354 & 0.9142 & 0.4610\\ 0.9106 & 0.5795 & 0.5666 & 0.5387 & 0.7092 & 1.0000 & 0.5795 & 0.5853 & 0.3592 \end{array}\right]$$

$$\gamma_{0\text{i}}=\left[\begin{array}{ccc} 0.7197 & 0.7049 & 0.6476 \end{array}\right]$$

### Application analysis

With the development of society, new buildings tend to reach saturation within a certain period. As existing buildings continue to age, renovation will become the primary engineering application. In recent years, Beijing has launched multiple batches of comprehensive renovation projects for old residential areas of state-owned enterprises. The third batch alone includes 924 residential areas (projects), covering 10.72 million square meters and involving 3,007 buildings. Assuming an average cost of 200 yuan per square meter for exterior wall renovation—this is a rough estimate based on various materials and processes, including labor and material costs—the cost of renovating the exterior walls of this batch of old residential areas is approximately 2.144 billion yuan. Over the next five years, if we calculate based on the average annual renovation of the exterior walls of similarly sized old residential areas, the budget for renovating these walls may exceed 2 billion yuan per year, resulting in a total budget of over 10 billion yuan for five years. However, in reality, in addition to old residential areas, there are also a large number of public and commercial buildings that may require exterior wall renovations, so the overall budget will undoubtedly far exceed this estimate.

The interfacial agent modified by PTB emulsion will play a significant role in this engineering application. IAWT demonstrates excellent bonding performance, particularly high early bond strength (Fig 3(a)). Additionally, the bond performance of IAWT remains strong in freeze-thaw and wind vibration environments (Fig 3(b)), enhancing its reliability in harsh conditions. Its favorable compression ratio and low water absorption (Fig 4) contribute to its flexibility and waterproofing, allowing it to adapt to changes in temperature and humidity. Furthermore, IAWT offers several environmental and economic advantages in construction, including:

(1) It roughens the surface of the original tiles without causing damage, allowing for the direct application of various decorative layers.
(2) It saves manpower and material resources, significantly reducing the construction period.
(3) It minimizes dust and noise hazards, generating environmental benefits.
(4) The use of IAWT extends the service life of buildings.
(5) IAWT with a P/W ratio of 1:3 exhibits excellent performance (Figs 10–12), greatly reducing the amount of original emulsion required, thus enhancing its economic viability.

## Conclusions

In this article, experimental tests are conducted to ensure the safe and reliable application of IAWT. The primary research focuses on the bond strength of IAWT in normal environments, bond strength under freeze-thaw cycles, bond strength after wind vibration, compressive-flexural ratio, water absorption, and microstructure, among other factors. The main conclusions are as follows:

(1) At a P/W ratio of 1:1, the bond strengths at 7 days and 14 days are 0.85 MPa and 0.88 MPa, respectively. After freeze-thaw cycling and wind vibration, the bond strengths are 0.56 MPa and 0.92 MPa, respectively. The compressive-flexural ratio is 4.84, and the water absorption is 0.85%.
(2) The addition of PTB emulsion enhances the bond strength of the interfacial agent. Cement hydration becomes more thorough, resulting in an increase in the micropores of the interfacial agent. The toughness of the interfacial agent is improved, and its crack resistance is enhanced. Simultaneously, its waterproof performance is also improved.
(3) As the content of PTB emulsion increases, IAWT develops an increasingly dense network structure. The internal hydration of IAWT is sufficient to generate a large number of micropores, and the cross-linked three-dimensional interpenetrating structure becomes more uniform and ordered. The network structure of ternary polymers is denser and more stable than that of single and binary polymers, resulting in higher strength of the cement paste.

Although this study conducted in-depth research on bond strength, it did not develop a predictive model. Future research could focus on the relationship between the type and amount of gel and the pore structure to establish a calculation model for bond strength.
